# *Nodes-and-connections* RNAi knockdown screening: identification of a signaling molecule network involved in fulvestrant action and breast cancer prognosis

**DOI:** 10.1038/oncsis.2015.32

**Published:** 2015-10-19

**Authors:** N Miyoshi, B S Wittner, K Shioda, T Hitora, T Ito, S Ramaswamy, K J Isselbacher, D C Sgroi, T Shioda

**Affiliations:** 1 Center for Cancer Research, Massachusetts General Hospital and Harvard Medical School, Charlestown, MA, USA; 2 Department of Surgery, Rinku General Medical Center, Osaka, Japan; 3 Molecular Pathology, Massachusetts General Hospital and Harvard Medical School, Charlestown, MA, USA

## Abstract

Although RNA interference (RNAi) knockdown screening of cancer cell cultures is an effective approach to predict drug targets or therapeutic/prognostic biomarkers, interactions among identified targets often remain obscure. Here, we introduce the *nodes-and-connections* RNAi knockdown screening that generates a map of target interactions through systematic iterations of *in silico* prediction of targets and their experimental validation. An initial RNAi knockdown screening of MCF-7 human breast cancer cells targeting 6560 proteins identified four signaling molecules required for their fulvestrant-induced apoptosis. Signaling molecules physically or functionally interacting with these four *primary node* targets were computationally predicted and experimentally validated, resulting in identification of four *second-generation node*s. Three rounds of further iterations of the prediction–validation cycle generated third, fourth and fifth generation of nodes, completing a 19-node interaction map that contained three predicted nodes but without experimental validation because of technical limitations. The interaction map involved all three members of the death-associated protein kinases (DAPKs) as well as their upstream and downstream signaling molecules (calmodulins and myosin light chain kinases), suggesting that DAPKs play critical roles in the cytocidal action of fulvestrant. The *in silico* Kaplan–Meier analysis of previously reported human breast cancer cohorts demonstrated significant prognostic predictive power for five of the experimentally validated nodes and for three of the prediction-only nodes. Immunohistochemical studies on the expression of 10 nodal proteins in human breast cancer tissues not only supported their prognostic prediction power but also provided statistically significant evidence of their synchronized expression, implying functional interactions among these nodal proteins. Thus, the *Nodes-and-Connections* approach to RNAi knockdown screening yields biologically meaningful outcomes by taking advantage of the existing knowledge of the physical and functional interactions between the predicted target genes. The resulting interaction maps provide useful information on signaling pathways cooperatively involved in clinically important features of the malignant cells, such as drug resistance.

## Introduction

Approximately 70% of naive primary breast cancers express estrogen receptor-α (ERα) and require estrogens for their growth and survival.^1^ Endocrine therapy suppresses estrogen-dependent proliferation of breast cancer cells and induces their apoptosis by reducing the supply of circulating estrogens and/or estrogen-induced intracellular signaling.^2, 3^ Although endocrine therapy has been proven beneficial, its clinical effectiveness is limited by *de novo* and acquired drug resistance.^4, 5^ To improve the long-term therapeutic outcome of estrogen-dependent breast cancer, elucidation of the molecular mechanisms of endocrine therapy resistance is urgently desired.^4, 6, 7^

Interactions between the estrogen signaling pathway and a wide variety of other intracellular signaling molecules affect breast cancer cell sensitivity to endocrine therapy.^8^ Ligand-activated ERα functions as a transcription factor that interacts with a large number of coregulator proteins and other transcription factors.^9, 10^ Activated ERα also initiates rapid intracellular signaling through interactions with growth factor signaling molecules at the plasma membrane.^4, 11, 12, 13^ Interactions of ERα with other signaling molecules affect functions of growth factor-activated protein kinases.^14, 15, 16, 17, 18, 19, 20^ The interferon-γ and the HER2/ERBB2-mitogen-activated protein kinase (MAPK) signaling pathways have been reported to play a pivotal role in antiestrogen resistance.^21, 22, 23^ These observations suggest that a highly complex network comprising many intracellular signaling molecules is involved in the development of resistance to endocrine therapy.

RNA interference (RNAi) knockdown screening of cell culture models is a powerful approach for identifying molecules involved in drug resistance.^24, 25^ Comprehensive RNAi knockdown screenings covering all known protein-coding genes in the human genome may reveal a signaling network involved in breast cancer cell resistance to endocrine therapy. However, genome-wide RNAi knockdown screening experiments are technically demanding,^24^ and integration of the screening results into a biologically informative signaling network is often challenging. To overcome such limitations of genome-wide RNAi knockdown screenings, we propose the use of *nodes-and-connections* RNAi knockdown screening. This approach starts with a small number of known positive-hit RNAi targets that are designated as the *primary node* molecules. Then, using bioinformatics tools, molecules that may functionally or physically interact with the primary nodes are predicted, and RNAi knockdown experiments focusing on these predicted node molecules validate them to identify the *secondary node* molecules. By iterating this prediction–validation cycle, deeper levels of nodes are progressively determined, eventually resulting in the generation of a comprehensive molecular interaction map connecting most, if not all, the primary nodes. A small number of nodes whose involvement in the network is predicted but cannot be experimentally validated because of technical limitations are allowed to be included in the molecular interaction map for effective and flexible prediction of practically useful signaling networks.

In the present study, we apply the *nodes-and-connections* RNAi knockdown screening approach to generate an interaction map of molecules necessary for the fulvestrant-induced MCF-7 cell apoptosis. The resulting interaction map reveals the critical importance of the death-associated protein kinase (DAPK) family of pro-apoptotic signaling kinases as well as their downstream effectors, including STAT3 (signal transducer and activator of transcription 3) and myosin light chains. The *in silico* Kaplan–Meier survival analysis reveals that not only experimentally validated nodes but also nodes without validation in the interaction map yield promising prognostic biomarkers predicting recurrence of breast cancer. Immunohistochemical evaluation of the nodal protein expression in human breast cancer tissues supports their prognostic predictive power, and statistically significant evidence is presented that these nodal proteins are expressed in a highly synchronized manner, implying organized regulation of their expression. These results demonstrate the usefulness of the *nodes-and-connections* RNAi knockdown screening for rapid and cost-effective identification of clinically relevant sets of biomarkers and drug targets using cell culture systems.

## Results

### Mapping interactions between the signaling molecules required for fulvestrant-induced MCF-7 cell death by the *nodes-and-connections* RNAi knockdown screenings

**Figure 1:**
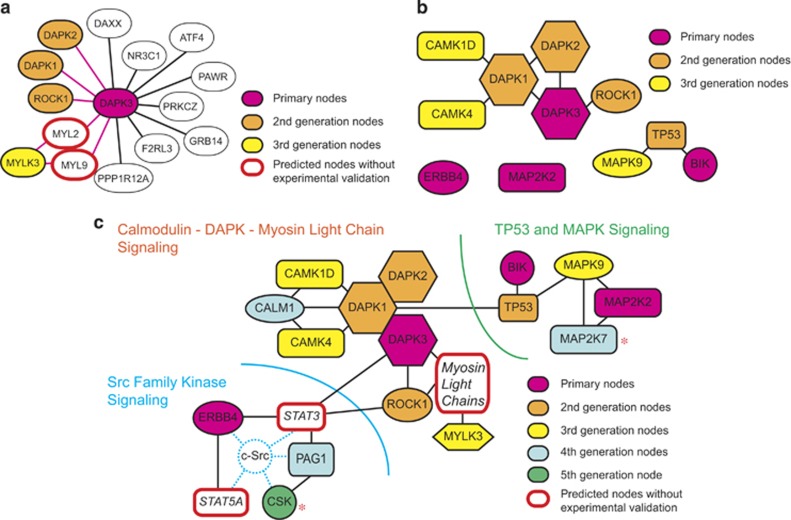
The *nodes-and-connections* strategy generating an interaction map of signaling molecule network required for the fulvestrant-induced MCF-7 cell apoptosis. (**a**) The *in silico* prediction and experimental validation of proteins functionally and/or physically interacting with the DAPK3 protein kinase (red). The Ingenuity Pathway Analysis (IPA) and other bioinformatics tools predicted 13 proteins regulating DAPK3 function or being regulated by it. Among them, requirement of three protein kinases (DAPK1, DAPK2 and ROCK1; orange) for the fulvestrant-induced MCF-7 cell apoptosis was confirmed by RNAi knockdown experiments. Although requirement of two other protein kinases (MYL2 and MYL9; open red) was unable to be confirmed by RNAi knockdown for technical limitations, requirement of MYLK3 (yellow), which regulates MYL2 and MYL9, was experimentally validated. RNAi knockdown of the other eight proteins (white) did not affect the fulvestrant-induced MCF-7 cell apoptosis. (**b**) An intermediate interaction map consisting of experimentally validated primary, second-generation and third-generation nodes. Two primary nodes (ERBB4 and MAPK2) are not yet connected to any other nodes. The four primary nodes have not been connected yet. (**c**) The interaction map of signaling molecules required for the fulvestrant-induced MCF-7 cell apoptosis. Three nodes (Myosin light chains, STAT3 and STAT5A) in this map were predicted by *in silico* analyses but not experimentally validated. All primary nodes are connected to each other, directly or indirectly, and the entire map is roughly divided into three sections—namely, SFK (Src family protein tyrosine kinase) signaling, DAPK signaling and other signaling molecules including BIK, TP53, MAP2Ks and MAPKs. Requirement of MAP2K7 and CSK (*) was also identified in an independently performed kinome-wide RNAi knockdown screening.

Our previous studies have shown that fulvestrant kills estrogen-dependent MCF-7 human breast cancer cells by inducing apoptosis in a manner dependent on the TP53 tumor suppressor protein and the BIK pro-apoptotic member of the BCL2 family of apoptosis regulators.^26, 27^ Fulvestrant-induced expression of BIK mRNA requires wild-type TP53 that is strongly expressed in MCF-7 cells.^26, 27^ To investigate the signaling network required for the fulvestrant-induced MCF-7 cell death in more detail, we performed RNAi knockdown screenings using 6560 arrayed short hairpin RNA (shRNA) expression plasmids targeting the human kinome,^28^ cell cycle proteins and apoptosis regulators. This preliminary screening yielded four signaling molecules—namely, BIK, ERBB4, DAPK3 and MAP2K2. Signaling by ERBB4 (also known as HER4), a member of the ERBB/HER family receptor kinases, promotes differentiation and growth inhibition of breast cancer cells.^29^ Whereas expression of the other three members of the ERBB/HER family in breast cancer is strongly linked to poor prognosis, increased expression of ERBB4 is more consistently correlated with a favorable prognosis.^29^ DAPK3 (also known as ZIP kinase (ZIPK)) is a member of the DAPK family pro-apoptotic signaling kinases.^30, 31^ The mitogen-activated protein kinase kinase 2 (MAP2K2 or MEK2) regulates the MAPK/ERK protein kinases that directly phosphorylate multiple nuclear proteins involved in cell cycle regulation.^32^ Although these four signaling molecules did not have any known direct interactions among each other, we presumed that they might be parts of a large network of molecules required for the fulvestrant-induced MCF-7 cell death. To identify this hypothetical network, we extended our RNAi knockdown screenings with the *nodes-and-connections* approach, as outlined in Supplementary Figure 1. In this study, the four signaling molecules identified above are designated as the *primary nodes* (Figure 1b).

To initiate the *nodes-and-connections* screening, molecules directly or indirectly interacting with the primary nodes were predicted using the Ingenuity Pathway Analysis.^33^ For example, DAPK3 was predicted to interact with 13 proteins: DAPK1, DAPK2, ROCK1, MYL2, MYL9, DAXX, NR3C1, ATF4, PAWR, PRKCZ, GRB14, F2RL3 and PPP1R12A (Figure 1a). The computationally predicted molecules interacting with the primary nodes were subjected to RNAi knockdown using shRNA-expressing lentiviruses to test whether they are required for the fulvestrant-induced MCF-7 cell death. Among the 13 proteins predicted to interact with DAPK3, knockdown of three protein kinases DAPK1, DAPK2, and ROCK1 resulted in strong fulvestrant resistance (Figure 1a and Supplementary Figure 2). DAPK1, DAPK2 and DAPK3 belong to the DAPK family of pro-apoptotic protein kinases.^30, 31, 34, 35^ Reduced expression of DAPK1 mRNA in human breast cancer correlates with a poor prognosis.^36, 37^ DAPK1 activates DAPK3 to amplify apoptotic signals.^38^ DAPK1, DAPK2 and DAPK3 induce apoptosis when overexpressed in human cells,^30^ and they physically interact with each to form a multiprotein complex apoptosis inducer.^30, 31^ ROCK1 (Rho-associated coiled-coil containing protein kinase 1) phosphorylates DAPK3 to activate it,^39^ and both ROCK1 and DAPK3 phosphorylate myosin light chain subunits to increase the actin-activated myosin ATPase activity.^40^ DAPK1, DAPK2 and ROCK1, which interact with the DAPK3 primary node, are the *second-generation nodes* in the molecular interaction map for the fulvestrant-induced MCF-7 cell death. Because RNAi knockdown of MYL2 or MYL9 myosin light chains strongly reduced MCF-7 cell viability even in the absence of fulvestrant,^41^ their requirement for the fulvestrant-induced apoptosis was not experimentally confirmed. However, RNAi knockdown of the MYLK3 myosin light chain kinase, which phosphorylates MYL2 and MYL9,^42^ did not damage MCF-7 cell viability and caused their fulvestrant resistance (Figure 1a). Because of the nonspecific cell toxicity of MYL2 or MYL9 knockdown, Supplementary Figure 2 presents data demonstrating fulvestrant resistance induced by MYLK3 knockdown. The molecular interaction map thus includes MYLK3 as an experimentally validated *third-generation node*, whereas its substrate *myosin light chains* are included as *predicted second-generation* nodes (Figure 1a).

Because our previous study showed that expression of BIK mRNA is dependent on TP53,^26^ TP53 is a second-generation node (Figure 1b). TP53 also plays critical roles in DAPK1-induced cell death,^30, 31^ and in human breast cancers the epigenetic suppression of DAPK1 expression and mutational inactivation of TP53 occur in a mutually exclusive manner,^43^ supporting the notion that DAPK inactivation in breast cancers might contribute to attenuation of TP53-dependent drug actions.

Computational prediction of molecules interacting with the second-generation nodes followed by experimental validation of their requirement for the fulvestrant-induced MCF-7 cell death with RNAi knockdown yielded three additional third-generation nodes—namely, the MAPK9 mitogen-activated protein kinase and the CAMK1D and CAMK4 calmodulin-dependent protein kinases (Figure 1b and Supplementary Figure 2). MAPK9 (also known as the JUN N-terminal kinase 2 or JNK2) blocks ubiquitination of TP53 to increase its stability.^44^ DAPK1, DAPK2, CAMK1D and CAMK4 are regulated by the Ca^2+^/calmodulin system,^31, 45^ and RNAi knockdown of a calmodulin CALM1 caused MCF-7 cell resistance to fulvestrant (Figure 1c and Supplementary Figure 2a). CALM1 is thus a *fourth-generation node*.

Extension from the MAP2K2 primary node and the MAPK9 third-generation node through an iteration of the computational prediction - RNAi validation cycle yielded MAP2K7 as a fourth-generation node (Figure 1c and Supplementary Figure 2a). Our previous study also detected the requirement of MAP2K7 for the fulvestrant-induced MCF-7 cell death.^46^ Furthermore, Iorns *et al.*^25^ demonstrated the requirement of MAP2K7 for inhibition of MCF-7 cell proliferation by tamoxifen, another antiestrogen drug prescribed for ERα-positive breast cancers. Independent reidentification of MAP2K7 as a kinase required for antiestrogen action in MCF-7 cells supports the validity and efficiency of the *nodes-and-connections* approach.

Computationally predicted new nodes interacting with DAPK3, ERBB4 and ROCK1 included STAT3 that directly binds to the PAG1/CBP (CSK binding protein).^47^ Although RNAi knockdown of STAT3 was unsuccessful because of nonspecific cell damage, RNAi knockdown of PAG1 caused MCF-7 cell resistance to fulvestrant (Supplementary Figures 2a and b). PAG1 interacts with the CSK c-Src tyrosine kinase, a negative regulator of the c-Src oncoprotein and other Src family kinases,^48, 49^ and our previous study demonstrated requirement of CSK for the fulvestrant-induced MCF-7 cell death.^46^ Thus, PAG1 and CSK are *fourth- and fifth-generation nodes*, respectively, and STAT3 is a predicted node (Figure 1c). STAT5A, which is another ERBB4-interacting signaling molecule, is also included in the interaction map as a predicted node based on its close involvement in the c-Src signaling (Figure 1c). All the nodes in the map of molecular interactions required for the fulvestrant-induced MCF-7 cell death are now connected, forming a large network consisting of three major components—namely, the calmodulin–DAPK–myosin light chain signaling, the TP53–MAPK signaling and the Src family kinase signaling (Figure 1c). Table 1 lists the numbers of independent shRNA lentiviral clones that significantly suppressed the cytocidal action of fulvestrant in the present study (note that RNAi knockdown experiments for BIK^27^ and TP53^26^ previously described by us are not included in Table 1). Supplementary Table 1 lists The RNAi Consortium (TRC) clone ID and shRNA sequences used in the present RNAi knockdown screenings.

### Expression of the mRNA transcripts for the nodal genes is affected by 17β-estradiol and/or fulvestrant

**Figure 2:**
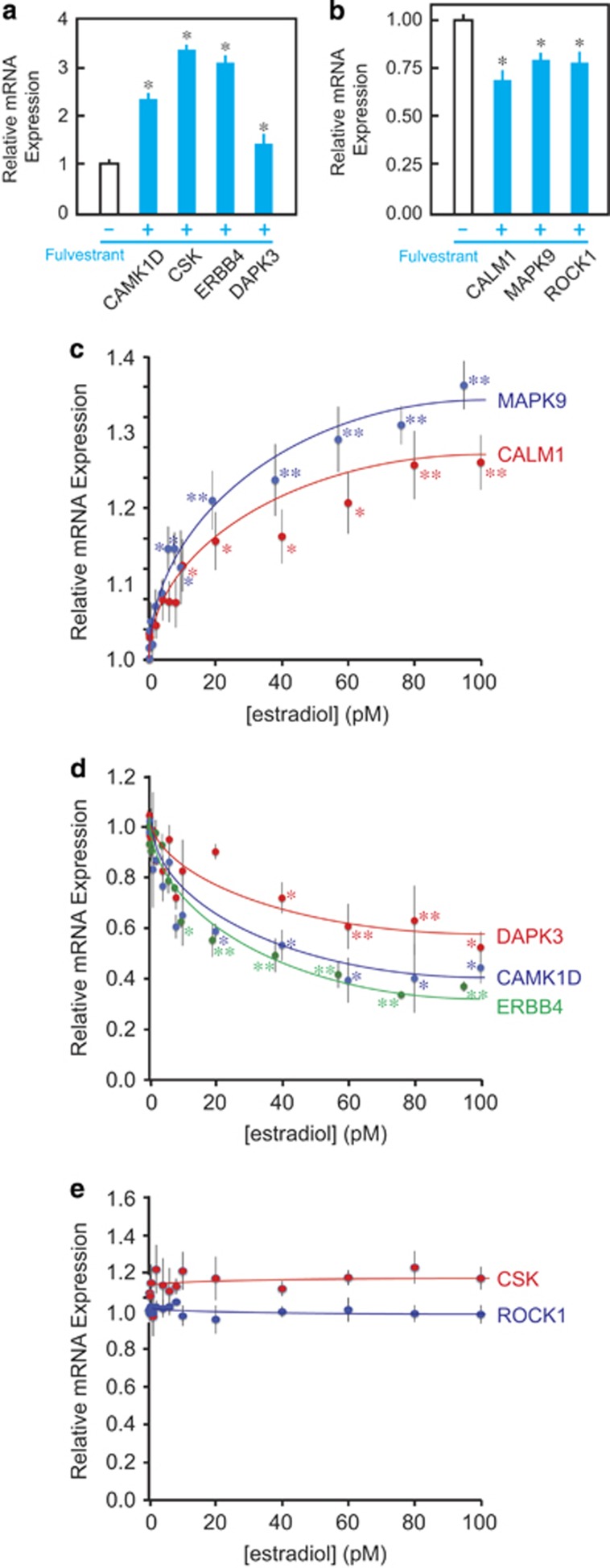
Effects of fulvestrant and 17β-estradiol on expression of the mRNA transcripts for the interaction map nodes in MCF-7 cells. (**a**, **b**) Cells were exposed to 100 nM fulvestrant or vehicle for 48 h followed by mRNA expression profiling by Affymetrix microarray. Robust Multi-array Average (RMA)-normalized relative mRNA expression of genes induced (**a**) or suppressed (**b**) by fulvestrant are shown; mRNA expression in vehicle-exposed cells is defined as 1.00 for each gene (mean±s.e.m., *n*≥5). Asterisk indicates statistically significant changes compared with exposure to vehicle (analysis of variance (ANOVA) *P<0.05). (**c–e**) Cells were exposed to varying concentrations of 17β-estradiol for 48 h followed by Affymetrix transcriptomal profiling. RMA-normalized relative mRNA expression of genes induced (**a**), suppressed (**b**) or unchanged (**c**) by 17β-estradiol are shown. Each datum point represents results of at least three independent experiments (mean±s.e.m.), and asterisk indicates statistically significant changes compared with exposure to vehicle (ANOVA *P<0.05; **P<0.005).

As fulvestrant kills MCF-7 cells by shutting down the estrogen-dependent cell survival signaling,^50^ we determined whether expression of the mRNA transcripts for the nodal genes is affected by ERα agonists and/or antagonists. Affymetrix microarray analysis (Santa Clara, CA, USA) revealed significant increase in mRNA expression for CAMK1D, CSK, ERBB4 and DAPK3 after exposure of MCF-7 cells to 100 nM fulvestrant for 48 h (Figure 2a). Under this experimental condition, intracellular ERα protein is completely degraded but cellular viability has not yet been significantly reduced.^26, 27, 46^ In contrast, mRNA expression for CALM1, MAPK9 and ROCK1 slightly decreased after exposure to fulvestrant (Figure 2b). After cells were exposed to varying concentrations of 17β-estradiol for 48 h, expression of *CALM1* and *MAPK9* mRNA was induced (Figure 2c), whereas *CAMK1D*, *DAPK3* and *ERBB4* mRNA expression was suppressed (Figure 2d). In contrast, mRNA expression of CSK or ROCK1 was not affected by exposure to 17β-estradiol (Figure 1e) despite their significant induction (Figure 1a) or suppression (Figure 1b) by fulvestrant, respectively. Thus, MAPK9, CALM1, DAPK3, CAMK1D and ERBB4 are estrogen-responsive genes in MCF-7 cells, whereas expression of CSK1 and ROCK1 is fulvestrant responsive but not estrogen responsive through unknown mechanisms.

### The *in silico* Kaplan–Meyer analysis suggests significant prediction power of the nodal genes as prognostic biomarkers of breast cancer

**Figure 3:**
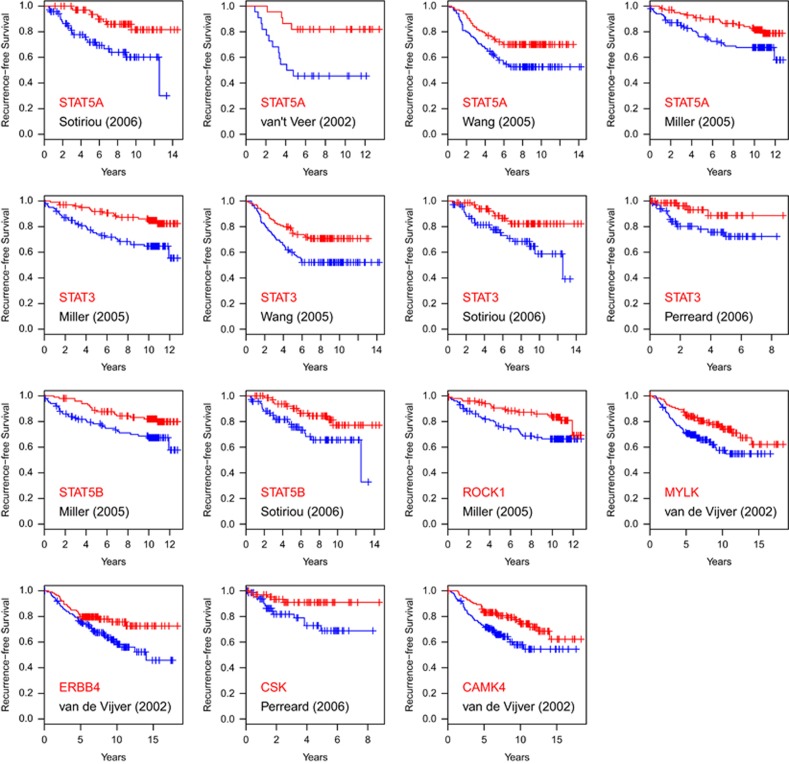
Kaplan–Meier recurrence-free survival curves of breast cancer cohorts whose surgically resected primary tumors strongly or weakly expressed the interaction map nodes. Each cohort of patients involved in the previously reported studies on breast cancer prognosis and mRNA expression profiles were divided into two groups of identical numbers of patients strongly (red) or weakly (blue) expressing the interaction map nodes. Thus, the weakest mRNA expression in a tumor belonging to the subcohort indicated by red line was stronger than the strongest mRNA expression in the subcohort indicated by blue line, although the absolute mRNA amounts and the shape of their distributions may differ for each panel. Kaplan–Meier curves of recurrence-free survival were drawn for each of the two groups, and the pair of curves showing significantly different survival rate are shown (false discovery rate (FDR) <0.25, Benjamini–Hochberg corrected one-sided log-rank *P*-values). Each panel indicates the pair of curves, the node protein and the cohort study.

To explore the relevance of the interaction map generated in this study to human breast cancer, we examined whether mRNA expression of the nodal genes in tumor tissues of previously published breast cancer cohorts correlates with better prognosis by *in silico* Kaplan–Meier analysis (Figure 3). Patients were ranked by mRNA expression for each nodal gene, and two independent recurrence-free survival curves were drawn for the top 50 percentile (high expressers, red curves) and bottom 50 percentile (low expressers, blue curves) patients. Log-rank testing identified three nodal genes (*STAT3*, *STAT5A* and *STAT5B*) whose stronger expression correlated with significantly better prognosis in multiple cohorts. Expression of four other nodal genes (*ROCK1*, *ERBB4*, *CSK* and *CAMK4*) and *MYLK*, encoding a myosin light chain isoform as the nodal gene *MYLK3*, also correlated with significantly better prognosis in a single cohort (data not shown). These results suggest that the interaction map of molecules required for fulvestrant-induced death of MCF-7 cell culture model may provide insights into clinically useful prognostic biomarkers of breast cancer.

### Strong protein expression of nodal molecules in human breast cancer tissues correlates with better prognosis

**Figure 4:**
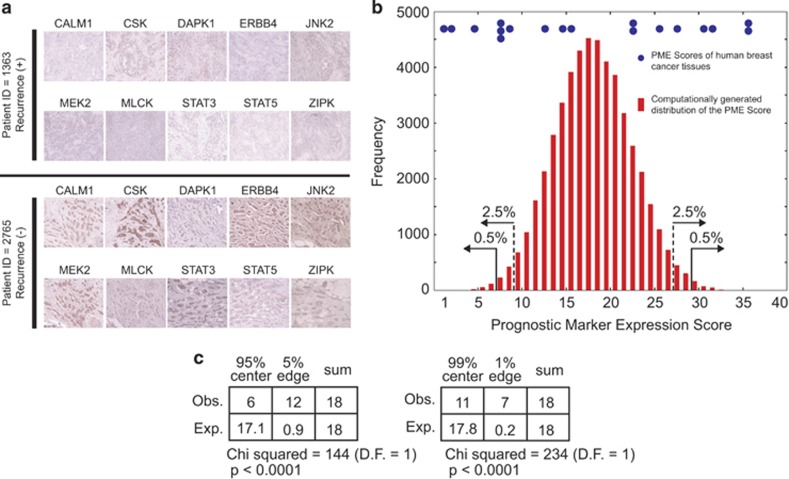
Expression of the interaction map node proteins in human breast cancer tissues. (**a**) Immunohistochemical staining of a recurrence-positive tumor (top) and a recurrence-negative tumor (bottom) for 10 selected interaction map node proteins. (**b**, **c**) A permutation test demonstrating synchronized expression of the 10 interaction map node proteins in human breast cancer tissues. The PME score is the sum of intensity scores of the 10 antigens (see Materials and methods for details). The histogram (**b**) shows a theoretical distribution of the PME score computationally generated by 50 000-cycle permutations of randomly selected intensity scores of the raw data, and arrows indicate boundaries of 5 or 1% two-tail extremities. The PME scores of the 18 tumor specimens are shown with blue dots (**b**), and their statistical significance was determined by the χ^2^ test (**c**).

To determine whether nodal protein expression in breast cancer tissues correlates with prognosis, paraffin-embedded pathological slides of surgically excised tumor tissues obtained from 18 age- and stage-matched breast cancer patients (clinical and pathological profiles shown in Supplementary Table 4) were subjected to immunohistochemical analysis for selected 10 nodal proteins. Nodal protein expression was scored to calculate the Prognosis Marker Expression (PME) score for each patient as described in the Materials and methods. Figure 4a shows immunohistochemical images of tumors with the lowest PME score (patient ID=1363, PME score=2.5, recurrence (+)) and the highest PME score (patient ID=2765, PME score=36, recurrence (−)) with the same T/N/M (tumor/node/metastasis) and clinical stages (4/1/0, IIIb). As shown in Supplementary Table 5, the PME scores of all patients who experienced breast cancer recurrence were significantly smaller than the scores of all patients without recurrence (*P*=0.0086). The PME scores were also significantly smaller for recurrent breast cancers when the same analysis was performed only with patients who received endocrine therapy (*P*=0.046), whereas no statistically significant recurrence-associated difference in the PME scores was observed with patients who did not receive endocrine therapy (*P*=0.124). These results suggest that expression profiling of the nodal proteins may be useful for predicting prognosis of breast cancer after endocrine therapy.

We next performed a permutation test to determine whether expression of the 10 nodal proteins is mutually independent. A bell-shaped distribution of the PME scores was generated by computational simulation without considering mutual effects of the nodal proteins on their expression (red bars). The χ^2^ tests indicated that the 18 PME scores of breast cancer patients (Figure 4b, blue dots) do not follow this simulated distribution (Figure 4c; *P*<0.0001 for both 5 and 1% tails), indicating that expression of each nodal protein is not mutually independent. The apparent associations among the nodal proteins for their expression in breast cancer tissues may imply that resistance to endocrine therapy might occur through a coordinated loss of multiple nodes in the interaction map rather than loss or dysfunction of a single nodal molecule. It is unknown as to whether suppression of a single nodal molecule in breast cancer *in vivo* is insufficient to cause clinically observed drug resistance that is in contrast to the MCF-7 cell culture model showing significant resistance after single molecule knockdown. Alternatively, the apparently coordinated loss of multiple nodal molecule *in vivo* might reflect an involvement of common mechanisms regulating their expression such as epigenetic suppression.

## Discussion

High-throughput knockdown/knockout screening of cell culture models is a powerful approach to identify molecules involved in drug resistance.^51, 52, 53^ Whereas a standard knockdown/knockout study performs screening data generation and bioinformatics data analysis as two separate stages, a *nodes-and-connections* approach performs multiple cycles of iteration of knockdown/knockout experiments and data analysis. This novel approach has the following merits.

First, even when experimental knockdown/knockout of certain nodes is technically difficult, such nodes can be considered *pending* and cautiously included in the molecular interaction map if results of later cycles of iteration or other studies support their validity, possibly reducing false negatives caused by technical limitations. Examples of such nodes in our present study are STAT3, STAT5A and myosin light chains (Figure 1c), whose validity was supported by *in silico* survival curve analysis (Figure 3) and immunohistochemical examinations of their usefulness as prognosis markers of breast cancer (Figure 4).

Second, data analysis is efficient because physical or functional interactions between nodes are already known. The interaction map generated in the present study suggests importance of TP53–MAPK, Calmodulin–DAPK–myosin light chain and Src Family Kinase signaling in the fulvestrant-induced MCF-7 cell death (Figure 1c). Fulvestrant may kill MCF-7 cells through coordinated effects via these signaling pathways, namely (1) TP53 induces the pro-apoptotic effector BIK while affecting cell cycle progression via MAPKs; (2) calmodulin-regulated DAPKs cause death signaling involving myosin light chains; and (3) ERBB4-STAT3/5A suppress the c-Src cell survival signal involving PAG1 and CSK.

Third, the size of a *nodes-and-connections* RNAi knockdown screening study is flexible. During the iterated prediction–validation cycles, the interaction map gradually develops from the unconnected primary nodes to form a relatively complex functional network involving multiple generations of nodes (Figure 1). Investigators can terminate the iteration when a sufficient amount of information has been obtained to depict a closed interaction map and/or to generate testable new hypotheses. When newly predicted genes are no longer experimentally validated positive after several cycles of iteration, the screening may be coming closer to saturation. This is in contrast to the standard screening approach that does not provide real-time opportunities to evaluate possible screening saturation. In the *nodes-and-connections* screening, investigators may also have opportunities to evaluate the validity of an ongoing screening experiment by testing whether it is detecting known positive genes. For example, Iorns performed a small interfering RNA-based knockdown screening of the 779-gene human kinome and identified CDK10, CRK7 and MAP2K7 as signaling kinases required for inhibition of MCF-7 cell proliferation by tamoxifen,^22, 25^ and our present screening successfully ‘rediscovered' MAP2K7 as a kinase required for fulvestrant-induced MCF-7 cell death (Figure 1c and Supplementary Figure 2), suggesting the importance of MAP2K7 in estrogen signaling in this cell line.

Taking advantage of the ‘expressome' database of estrogen dose-dependent transcriptomal changes in MCF-7 cells developed in our previous study,^54^ we characterized estrogen and fulvestrant responsiveness of the nodal genes (Figure 2). We identified CAMK1D, CSK, ERBB4 and DAPK3 as fulvestrant-induced genes (Figure 2a), and three of them (CAMK1D, ERBB4 and DAPK3) indeed showed estradiol dose-dependent suppression (Figure 2d). Similarly, CALM1, MAPK9 and ROCK1 were fulvestrant-suppressible genes (Figure 2b), and two of them (CALM1 and MAPK9) were also estrogen inducible (Figure 2c). Interestingly, despite the significant fulvestrant effects on mRNA expression of CSK and ROCK1, these genes did not respond to any concentration of estradiol (Figure 2e), suggesting the existence of fulvestrant-dependent transcriptional regulating mechanism independent of estrogen signaling. Further studies will be required to explain the differences in the fulvestrant and estradiol effects on expression of the CSK and ROCK1 mRNA transcripts.

The clinical relevance of the outcome of the present study has been demonstrated by the *in silico* Kaplan–Meier analysis of breast cancer cohorts (Figure 3) and immunohistochemical examination of expression of the nodes in breast cancer tissues (Figure 4). In agreement with previously published studies,^29^ stronger expression of ERBB4 in breast cancer cohorts correlated with better prognosis (Figure 3). Similarly, stronger expression of STAT3, STAT5A and STAT5B, which functionally interact with ERBB4, also correlated with better prognosis. In addition, expression of ROCK1, CSK, CAMK4 and myosin light chain kinase also showed significant prognostic prediction power. These results demonstrate that expression of the nodes, whether experimentally validated or included in the interaction map as predicted nodes, may be useful as clinically relevant biomarkers. The potential usefulness of the nodes as prognostic biomarkers has also been supported by immunohistochemical examinations of their expression in breast cancer, in which stronger expression correlated with recurrence-free status of breast cancer patients who received endocrine therapy (Figure 4 and Supplementary Table 5). Such correlation was not statistically significant for patients who did not receive endocrine therapy, whereas it was significant for the entire patients combined with or without endocrine therapy (Supplementary Table 5). Interestingly, a permutation test demonstrated significant mutual association among expression of the nodes—that is, breast cancers tend to express the nodes in a coordinated manner (all nodes are strongly expressed, or no node is detected). This observation may imply a possibility that fulvestrant resistance of breast cancer might *not* be caused by loss of one or a few components of the interaction map. Instead, many of the components of the network could be lost simultaneously in the endocrine therapy-resistant tumors. Future studies should address the question of whether expression of multiple nodes is regulated by a common mechanism that could be involved in the mechanism of acquired resistance to endocrine therapy.

Because the *nodes-and-connections* RNAi knockdown screening approach is dependent on the existing knowledge on the nodes, it may be associated with ‘subjective bias' introduced during selections of the screening targets. Therefore, this approach might not be ideal for studies that require strictly unbiased screening conditions such as toxicological assessments for regulatory purposes. Because of its dependence on the existing knowledge, it may have limited power to discover unknown molecular interactions. Nonetheless, the *nodes-and-connections* approach still has significant discovery power within the contexts of known signaling pathways and molecular interactions and is appropriate for obtaining useful mechanistic insights into biologically or clinically important phenomena such as drug resistance. Thus, the *nodes-and-connections* screening and the genome-wide thorough screening are designed to aim distinct goals. These two approaches should be compared based on the benefits and limitations depending on the priorities of the screening projects.

In summary, the *nodes-and-connections* RNAi knockdown screening approach efficiently generates a comprehensive map of functional interactions between genes involved in a specific cellular phenotype. In this approach, one cycle of iteration consists of computational prediction of relatively small numbers of knockdown targets based on known positive target genes and their experimental validation by RNAi knockdown, and the cycle is repeated until sufficient amount of information is obtained from the interaction map. With this approach, a knockdown screening study can reduce false positives and adjust its size during the ongoing screening operation, and interpretation of the biological significance of its outcome is straightforward. The *nodes-and-connections* screening thus provide unique opportunities to take advantage of the database-stored knowledge of functional molecular interactions to enhance the efficiency of RNAi knockdown screening.

## Materials and methods

### Cell culture

MCF-7 human breast cancer cells (BUS stock) were provided by C Sonnenschein and Ana M Soto (Tufts University, Medford, MA, USA),^55^ and its fulvestrant-sensitive monoclonal subline W2 has been described in our previous study.^50^ Cells were maintained in Dulbecco's modified Eagle's medium supplemented with 5% fetal calf serum (HyClone, DEFINED grade; Thermo Scientific, Waltham, MA, USA) in 10% CO_2_ at 37 °C. Research-grade fulvestrant was purchased from Tocris (Ellisville, MO, USA). Fulvestrant-induced W2 cell apoptosis was evaluated by crystal violet staining after exposure to 100 nM fulvestrant for 7 days in the presence of 10% nonstripped fetal calf serum. Detailed method of cell viability analysis has been described in our previous paper.^46^

Following the criteria adopted in the original study describing the development and use of the TRC shRNA lentiviral libraries,^28^ our RNAi knockdown screening experiments defined *positive hit* as a target with at least two distinct shRNA lentiviral clones inducing a statistically significant resistance to fulvestrant compared with cells infected with the pLKO.1 control virus (*P*<0.05, analysis of variance). Because distinct shRNAs are expected to have different spectra of off-target effects, this criterion is expected to filter out off-target effects. For some positive-hit targets, fulvestrant resistance was observed with only two or three shRNA lentiviral clones among five or larger numbers of viral clones. This incompleteness reflects a commonly known technical limitation of the RNAi knockdown experiments using the TRC shRNA lentiviral library that was thoroughly evaluated in its original literature.^28^

### RNAi knockdown

Lentiviruses expressing shRNA species targeting human mRNA transcripts were produced using the pLKO.1 vector harboring a puromycin resistance marker gene as previously described.^28^ An arrayed shRNA lentivirus library targeting human kinome was prepared by transfecting HEK293T packaging cells with TRC library of 6560 kinome shRNA hairpin plasmids (Broad Institute, Cambridge, MA, USA) together with the pMD2G expression plasmid for vesicular stomatitis virus glycoprotein and the pCMV-dR8.91 plasmid for the core lentiviral genome. MCF-7 cells grown at 5–10 × 10^4^ cells/well density were infected with 4–8 multiplicity of infection titers of lentiviruses in the presence of 8 μg/ml polybrene under 1200 *g* gravity for 60 min. Medium was changed 48 h after infection, and successfully infected cells were selected by exposure to 2.5 μg/ml puromycin for 48 h.

### Transcriptomal profiling

Transcriptomal profiles of MCF-7 cells determined by Affymetrix microarray has been described in our previous studies.^50, 54^ Microarray data of MCF-7 cells exposed to fulvestrant and 17β-estradiol are available from the Gene Expression Omnibus (GEO) database of the National Center for Biotechnology Information (NCBI) accession numbers GSE14986 and GSE50705, respectively. The 17β-estradiol dose-dependent mRNA expression data are also retrievable from our website (http://mplwebserver.partners.org).

### Immunohistochemistry

Human breast cancer tissues were surgically excised from patients admitted in the Rinku General Medical Center (Osaka, Japan) between 2005 and 2008 and processed for the standard formalin fixation and paraffin embedding. Informed consent was obtained from the 18 patients involved in this study, whose clinical profiles are shown in Supplementary Table 4. The present study was approved in advance by the institutional review board of the Rinku General Medical Center. Immunohistochemical staining (hydrogen peroxide-diaminobenzidine chromogen system) was performed in the Special Pathology Core Facility of the Dana-Farber/Harvard Cancer Center at the Massachusetts General Hospital (Charlestown, MA, USA). All antibodies used in this study were rabbit polyclonal antibodies obtained from Abcam (Cambridge, MA, USA): anti-calmodulin (cat. no. ab38590), anti-CSK (cat. no. ab151590), anti-DAPK1 (cat. no. ab109382), anti-DAPK3/ZIPK (cat. no. ab78943), anti-ERBB4 (cat. no. ab38158), anti-JNK2 (cat. no. ab78495), anti-MEK2 (cat. no. ab28834), anti-MYLK (cat. no. ab71826), anti-STAT3 (ab137803) and anti-STAT5 (cat. no. ab68465). To ensure the same staining condition, slides of all the 18 tumor tissues were subjected to immunohistochemical procedure in a single batch for each antibody. Signal intensities of the stained sections were independently scored by two examiners using an arbitrary scale defined for each antigen (0=no signal, 1=weak, 2=moderate, 3=strong, and 4=strongest signal within the 18 sections), and means of the two raw scores (Supplementary Table 5) were used for further analysis. Identities of the pathological slides were blinded during the scoring procedure to eliminate bias. The PME score was defined for each tumor as the sum of these averaged intensity scores calculated for all the 10 antigens.

### Bioinformatics and statistics

The *in silico* prediction of interactions between nodes was performed using the Ingenuity Pathway Analysis tool (Ingenuity Systems, Redwood City, CA, USA). Kaplan–Meier analysis of recurrence-free survival was performed using the ‘survival' package of the R programming language (https://cran.r-project.org/web/packages/survival/) and publicly available mRNA expression and survival data (Supplementary Table 2). For each gene selected for this analysis (Supplementary Table 3) and each data set, a one-sided log-rank test was performed against the alternative hypothesis that patients with expression of the gene above the median have better survival. The *P*-values were then corrected for multiple hypotheses testing by the Benjamini–Hochberg method. Resulting false discovery rate estimates were considered significant if below 0.25.

To generate a theoretical distribution of the PME scores, an intensity score was randomly sampled from the 18 scores of each antigen, and the sum of these scores for all the 10 antigens were calculated to obtain a theoretical PME score. To test a null hypothesis that expression of each antigen is mutually independent (that is, *not* synchronized), a distribution of 50 000 theoretical PME scores was computationally generated, and its 1% and 5% two-tail cutoff values were determined. If expression of the 10 antigens is mutually independent, the majority of the observed PME scores are expected to follow this theoretical distribution. The χ^2^ tests were performed to determine whether the observed PME scores follow such a theoretical distribution.

## Figures and Tables

**Table 1:** Summary of a *node-and-connection* RNAi knockdown screening for genes required for fulvestrant-induced apoptosis of MCF-7 cells

*Two positive shRNA lentivirus clones*							
*ACVR1B*	*CHKB*	*DGKB*	*KFZp686K16132*	*MAPK4*	*NME5*	*PKMYT1*	*ROS1*
*ACVRL1*	*CIB4*	*DMPK*	*KHK*	*MAPK6*	*NME6*	*PLK1*	*RPS6KA4*
*ADRBK1*	*CKC42BP*	*DNAJC6*	*KSR1*	*MGC16169*	*NUAK2*	*PRKAB2*	*SRPK3*
*ADRBK2*	*CKMT2*	*EPHA2*	*LOC392265*	*MGC42105*	*PDGFRL*	*PRKAG3*	*STK38*
*AKT2*	*CLK3*	*FASTKD3*	***MAP2K7***	*MINK1*	*PFKL*	*PRKAR1A*	*TSSK6*
*ALS2CR7*	*CSNK1E*	*FLT4*	*MAPK11*	*MPP7*	*PHKB*	*PRKAR1B*	*TTN*
*CAMK4*	***DAPK2***	*GRK1*	*MAPK12*	*MYO3B*	*PHKG2*	*PTK7*	*ULK1*
*CHEK1*	*DCAMKL1*	*GSK3A*	*MAPK13*	*NEK10*	*PIK3R2*	*RIOK2*	*YES1*

*Three positive shRNA lentivirus clones*							
***CALM1***	***DAPK3***	*EPHB2*	*LOC441655*	*MPP3*	***PAG1***	*TESK1*	
***CAMK1D***	*DGKG*	***ERBB4***	***MAP2K2***	***MYLK3***	***ROCK1***	*TLK2*	
***DAPK1***	*EPHA3*	*GUCY2D*	***MAPK9***	*RAGE*	*RPS6KB2*	*TNIK*	

*Four positive shRNA lentivirus clones*							
***CSK***							

Abbreviations: RNAi, RNA interference; shRNA, short hairpin RNA.

Genes included in the interaction map (Figure 1) are shown in bold.

## References

[bib1] 1Butt AJ, Sutherland RL, Musgrove EA. Live or let die: oestrogen regulation of survival signalling in endocrine response. Breast Cancer Res 2007; 9: 306. 17980055 10.1186/bcr1779 PMC2242668

[bib2] 2Sainsbury R. The development of endocrine therapy for women with breast cancer. Cancer Treat Rev 2013; 39: 507–517. 23102614 10.1016/j.ctrv.2012.07.006

[bib3] 3Biganzoli L, Wildiers H, Oakman C, Marotti L, Loibl S, Kunkler I et al. Management of elderly patients with breast cancer: updated recommendations of the International Society of Geriatric Oncology (SIOG) and European Society of Breast Cancer Specialists (EUSOMA). Lancet Oncol 2012; 13: e148–e160. 22469125 10.1016/S1470-2045(11)70383-7

[bib4] 4Massarweh S, Schiff R. Unraveling the mechanisms of endocrine resistance in breast cancer: new therapeutic opportunities. Clin Cancer Res 2007; 13: 1950–1954. 17404074 10.1158/1078-0432.CCR-06-2540

[bib5] 5Bedard PL, Freedman OC, Howell A, Clemons M. Overcoming endocrine resistance in breast cancer-are signal transduction inhibitors the answer? Breast Cancer Res Treat 2008; 108: 307–317. 18351454 10.1007/s10549-007-9606-8

[bib6] 6Anderson H, Bulun S, Smith I, Dowsett M. Predictors of response to aromatase inhibitors. J Steroid Biochem Mol Biol 2007; 106: 49–54. 17604158 10.1016/j.jsbmb.2007.05.024

[bib7] 7Beelen K, Zwart W, Linn SC. Can predictive biomarkers in breast cancer guide adjuvant endocrine therapy? Nat Rev Clin Oncol 2012; 9: 529–541. 22825374 10.1038/nrclinonc.2012.121

[bib8] 8Chang J, Fan W. Endocrine therapy resistance: current status, possible mechanisms and overcoming strategies. Anticancer Agents Med Chem 2013; 13: 464–475. 22931419

[bib9] 9Paech K, Webb P, Kuiper GG, Nilsson S, Gustafsson J, Kushner PJ et al. Differential ligand activation of estrogen receptors ERalpha and ERbeta at AP1 sites. Science 1997; 277: 1508–1510. 9278514 10.1126/science.277.5331.1508

[bib10] 10Kim K, Thu N, Saville B, Safe S. Domains of estrogen receptor alpha (ERalpha) required for ERalpha/Sp1-mediated activation of GC-rich promoters by estrogens and antiestrogens in breast cancer cells. Mol Endocrinol 2003; 17: 804–817. 12576490 10.1210/me.2002-0406

[bib11] 11Kahlert S, Nuedling S, van Eickels M, Vetter H, Meyer R, Grohe C. Estrogen receptor alpha rapidly activates the IGF-1 receptor pathway. J Biol Chem 2000; 275: 18447–18453. 10749889 10.1074/jbc.M910345199

[bib12] 12Razandi M, Pedram A, Park ST, Levin ER. Proximal events in signaling by plasma membrane estrogen receptors. J Biol Chem 2003; 278: 2701–2712. 12421825 10.1074/jbc.M205692200

[bib13] 13Chung YL, Sheu ML, Yang SC, Lin CH, Yen SH. Resistance to tamoxifen-induced apoptosis is associated with direct interaction between Her2/neu and cell membrane estrogen receptor in breast cancer. Int J Cancer 2002; 97: 306–312. 11774281 10.1002/ijc.1614

[bib14] 14Migliaccio A, Castoria G, Di Domenico M, De Falco A, Bilancio A, Auricchio F. Src is an initial target of sex steroid hormone action. Ann NY Acad Sci 2002; 963: 185–190. 12095943 10.1111/j.1749-6632.2002.tb04109.x

[bib15] 15Song RX, McPherson RA, Adam L, Bao Y, Shupnik M, Kumar R et al. Linkage of rapid estrogen action to MAPK activation by ERalpha-Shc association and Shc pathway activation. Mol Endocrinol 2002; 16: 116–127. 11773443 10.1210/mend.16.1.0748

[bib16] 16Gururaj AE, Rayala SK, Vadlamudi RK, Kumar R. Novel mechanisms of resistance to endocrine therapy: genomic and nongenomic considerations. Clin Cancer Res 2006; 12: 1001s–1007s. 16467116 10.1158/1078-0432.CCR-05-2110

[bib17] 17Rayala SK, den Hollander P, Balasenthil S, Yang Z, Broaddus RR, Kumar R. Functional regulation of oestrogen receptor pathway by the dynein light chain 1. EMBO Rep 2005; 6: 538–544. 15891768 10.1038/sj.embor.7400417 PMC1369089

[bib18] 18Wong CW, McNally C, Nickbarg E, Komm BS, Cheskis BJ. Estrogen receptor-interacting protein that modulates its nongenomic activity-crosstalk with Src/Erk phosphorylation cascade. Proc Natl Acad Sci USA 2002; 99: 14783–14788. 12415108 10.1073/pnas.192569699 PMC137496

[bib19] 19Sun M, Paciga JE, Feldman RI, Yuan Z, Coppola D, Lu YY et al. Phosphatidylinositol-3-OH Kinase (PI3K)/AKT2, activated in breast cancer, regulates and is induced by estrogen receptor alpha (ERalpha) via interaction between ERalpha and PI3K. Cancer Res 2001; 61: 5985–5991. 11507039

[bib20] 20Santen RJ, Song RX, Zhang Z, Yue W, Kumar R. Adaptive hypersensitivity to estrogen: mechanism for sequential responses to hormonal therapy in breast cancer. Clin Cancer Res 2004; 10: 337S–345S. 14734489 10.1158/1078-0432.ccr-031207

[bib21] 21Ning Y, Riggins RB, Mulla JE, Chung H, Zwart A, Clarke R. IFNgamma restores breast cancer sensitivity to fulvestrant by regulating STAT1, IFN regulatory factor 1, NF-kappaB, BCL2 family members, and signaling to caspase-dependent apoptosis. Mol Cancer Ther 2010; 9: 1274–1285. 20457620 10.1158/1535-7163.MCT-09-1169 PMC2925293

[bib22] 22Gutierrez MC, Detre S, Johnston S, Mohsin SK, Shou J, Allred DC et al. Molecular changes in tamoxifen-resistant breast cancer: relationship between estrogen receptor, HER-2, and p38 mitogen-activated protein kinase. J Clin Oncol 2005; 23: 2469–2476. 15753463 10.1200/JCO.2005.01.172

[bib23] 23Vinayak S, Carlson RW. mTOR inhibitors in the treatment of breast cancer. Oncology 2013; 27: 38–44, 46, 48 passim. 23461041

[bib24] 24Root DE, Hacohen N, Hahn WC, Lander ES, Sabatini DM. Genome-scale loss-of-function screening with a lentiviral RNAi library. Nat Methods 2006; 3: 715–719. 16929317 10.1038/nmeth924

[bib25] 25Iorns E, Turner NC, Elliott R, Syed N, Garrone O, Gasco M et al. Identification of CDK10 as an important determinant of resistance to endocrine therapy for breast cancer. Cancer Cell 2008; 13: 91–104. 18242510 10.1016/j.ccr.2008.01.001

[bib26] 26Hur J, Bell DW, Dean KL, Coser KR, Hilario PC, Okimoto RA et al. Regulation of expression of BIK proapoptotic protein in human breast cancer cells: p53-dependent induction of BIK mRNA by fulvestrant and proteasomal degradation of BIK protein. Cancer Res 2006; 66: 10153–10161. 17047080 10.1158/0008-5472.CAN-05-3696

[bib27] 27Hur J, Chesnes J, Coser KR, Lee RS, Geck P, Isselbacher KJ et al. The Bik BH3-only protein is induced in estrogen-starved and antiestrogen-exposed breast cancer cells and provokes apoptosis. Proc Natl Acad Sci USA 2004; 101: 2351–2356. 14983013 10.1073/pnas.0307337101 PMC356954

[bib28] 28Moffat J, Grueneberg DA, Yang X, Kim SY, Kloepfer AM, Hinkle G et al. A lentiviral RNAi library for human and mouse genes applied to an arrayed viral high-content screen. Cell 2006; 124: 1283–1298. 16564017 10.1016/j.cell.2006.01.040

[bib29] 29Koutras AK, Fountzilas G, Kalogeras KT, Starakis I, Iconomou G, Kalofonos HP. The upgraded role of HER3 and HER4 receptors in breast cancer. Crit Rev Oncol Hematol 2010; 74: 73–78. 19481955 10.1016/j.critrevonc.2009.04.011

[bib30] 30Gozuacik D, Kimchi A. DAPk protein family and cancer. Autophagy 2006; 2: 74–79. 17139808 10.4161/auto.2.2.2459

[bib31] 31Bialik S, Kimchi A. The DAP-kinase interactome. Apoptosis 2014; 19: 316–328. 24220855 10.1007/s10495-013-0926-3

[bib32] 32Fremin C, Meloche S. From basic research to clinical development of MEK1/2 inhibitors for cancer therapy. J Hematol Oncol 2010; 3: 8. 20149254 10.1186/1756-8722-3-8 PMC2830959

[bib33] 33Vidal M, Cusick ME, Barabasi AL. Interactome networks and human disease. Cell 2011; 144: 986–998. 21414488 10.1016/j.cell.2011.02.016 PMC3102045

[bib34] 34Usui T, Okada M, Yamawaki H. Zipper interacting protein kinase (ZIPK): function and signaling. Apoptosis 2014; 19: 387–391. 24193917 10.1007/s10495-013-0934-3

[bib35] 35Isshiki K, Matsuda S, Tsuji A, Yuasa K. cGMP-dependent protein kinase I promotes cell apoptosis through hyperactivation of death-associated protein kinase 2. Biochem Biophys Res Commun 2012; 422: 280–284. 22580283 10.1016/j.bbrc.2012.04.148

[bib36] 36Shiloh R, Bialik S, Kimchi A. The DAPK family: a structure-function analysis. Apoptosis 2014; 19: 286–297. 24220854 10.1007/s10495-013-0924-5

[bib37] 37Michie AM, McCaig AM, Nakagawa R, Vukovic M. Death-associated protein kinase (DAPK) and signal transduction: regulation in cancer. FEBS J 2010; 277: 74–80. 19878310 10.1111/j.1742-4658.2009.07414.x

[bib38] 38Shani G, Marash L, Gozuacik D, Bialik S, Teitelbaum L, Shohat G et al. Death-associated protein kinase phosphorylates ZIP kinase, forming a unique kinase hierarchy to activate its cell death functions. Mol Cell Biol 2004; 24: 8611–8626. 15367680 10.1128/MCB.24.19.8611-8626.2004 PMC516725

[bib39] 39Hagerty L, Weitzel DH, Chambers J, Fortner CN, Brush MH, Loiselle D et al. ROCK1 phosphorylates and activates zipper-interacting protein kinase. J Biol Chem 2007; 282: 4884–4893. 17158456 10.1074/jbc.M609990200

[bib40] 40Walsh MP. Vascular smooth muscle myosin light chain diphosphorylation: mechanism, function, and pathological implications. IUBMB Life 2011; 63: 987–1000. 21990256 10.1002/iub.527

[bib41] 41Zhang C, Luo X, Liu L, Guo S, Zhao W, Mu A et al. Myocardin-related transcription factor A is up-regulated by 17beta-estradiol and promotes migration of MCF-7 breast cancer cells via transactivation of MYL9 and CYR61. Acta Biochim Biophys Sin 2013; 45: 921–927. 24084383 10.1093/abbs/gmt104

[bib42] 42Chan JY, Takeda M, Briggs LE, Graham ML, Lu JT, Horikoshi N et al. Identification of cardiac-specific myosin light chain kinase. Circ Res 2008; 102: 571–580. 18202317 10.1161/CIRCRESAHA.107.161687 PMC2504503

[bib43] 43Lehmann U, Celikkaya G, Hasemeier B, Langer F, Kreipe H. Promoter hypermethylation of the death-associated protein kinase gene in breast cancer is associated with the invasive lobular subtype. Cancer Res 2002; 62: 6634–6638. 12438260

[bib44] 44Fuchs SY, Adler V, Buschmann T, Yin Z, Wu X, Jones SN et al. JNK targets p53 ubiquitination and degradation in nonstressed cells. Genes Dev 1998; 12: 2658–2663. 9732264 10.1101/gad.12.17.2658 PMC317120

[bib45] 45Schumacher AM, Schavocky JP, Velentza AV, Mirzoeva S, Watterson DM. A calmodulin-regulated protein kinase linked to neuron survival is a substrate for the calmodulin-regulated death-associated protein kinase. Biochemistry 2004; 43: 8116–8124. 15209507 10.1021/bi049589v

[bib46] 46Yeh WL, Shioda K, Coser KR, Rivizzigno D, McSweeney KR, Shioda T. Fulvestrant-induced cell death and proteasomal degradation of estrogen receptor alpha protein in MCF-7 cells require the CSK c-Src tyrosine kinase. PLoS ONE 2013; 8: e60889. 23593342 10.1371/journal.pone.0060889 PMC3617152

[bib47] 47Hrdinka M, Horejsi V. PAG–a multipurpose transmembrane adaptor protein. Oncogene 2014; 33: 4881–4892. 24213579 10.1038/onc.2013.485

[bib48] 48Brdicka T, Pavlistova D, Leo A, Bruyns E, Korinek V, Angelisova P et al. Phosphoprotein associated with glycosphingolipid-enriched microdomains (PAG), a novel ubiquitously expressed transmembrane adaptor protein, binds the protein tyrosine kinase csk and is involved in regulation of T cell activation. J Exp Med 2000; 191: 1591–1604. 10790433 10.1084/jem.191.9.1591 PMC2213442

[bib49] 49Okada M. Regulation of the SRC family kinases by Csk. Int J Biol Sci 2012; 8: 1385–1397. 23139636 10.7150/ijbs.5141 PMC3492796

[bib50] 50Coser KR, Wittner BS, Rosenthal NF, Collins SC, Melas A, Smith SL et al. Antiestrogen-resistant subclones of MCF-7 human breast cancer cells are derived from a common monoclonal drug-resistant progenitor. Proc Natl Acad Sci USA 2009; 106: 14536–14541. 19706540 10.1073/pnas.0907560106 PMC2732824

[bib51] 51Wang T, Wei JJ, Sabatini DM, Lander ES. Genetic screens in human cells using the CRISPR-Cas9 system. Science 2014; 343: 80–84. 24336569 10.1126/science.1246981 PMC3972032

[bib52] 52Mohr SE, Smith JA, Shamu CE, Neumuller RA, Perrimon N. RNAi screening comes of age: improved techniques and complementary approaches. Nat Rev Mol Cell Biol 2014; 15: 591–600. 25145850 10.1038/nrm3860 PMC4204798

[bib53] 53Shalem O, Sanjana NE, Hartenian E, Shi X, Scott DA, Mikkelsen TS et al. Genome-scale CRISPR-Cas9 knockout screening in human cells. Science 2014; 343: 84–87. 24336571 10.1126/science.1247005 PMC4089965

[bib54] 54Shioda T, Rosenthal NF, Coser KR, Suto M, Phatak M, Medvedovic M et al. Expressomal approach for comprehensive analysis and visualization of ligand sensitivities of xenoestrogen responsive genes. Proc Natl Acad Sci USA 2013; 110: 16508–16513. 24062438 10.1073/pnas.1315929110 PMC3799318

[bib55] 55Soto AM, Sonnenschein C, Chung KL, Fernandez MF, Olea N, Serrano FO. The E-SCREEN assay as a tool to identify estrogens: an update on estrogenic environmental pollutants. Environ Health Perspect 1995; 103(suppl 7): 113–122. 10.1289/ehp.95103s7113 PMC1518887 8593856

