# Supplementary material for: Nodes-and-connections RNAi knockdown screening: identification of a signaling molecule network involved in fulvestrant action and breast cancer prognosis
Source: Oncogenesis. 2015 Oct 19;4(10):e172–. doi: 10.1038/oncsis.2015.32 (PMC4632093; doi:10.1038/oncsis.2015.32)
Supplement: Supplementary Figure 1 [file oncsis201532x1.pdf]

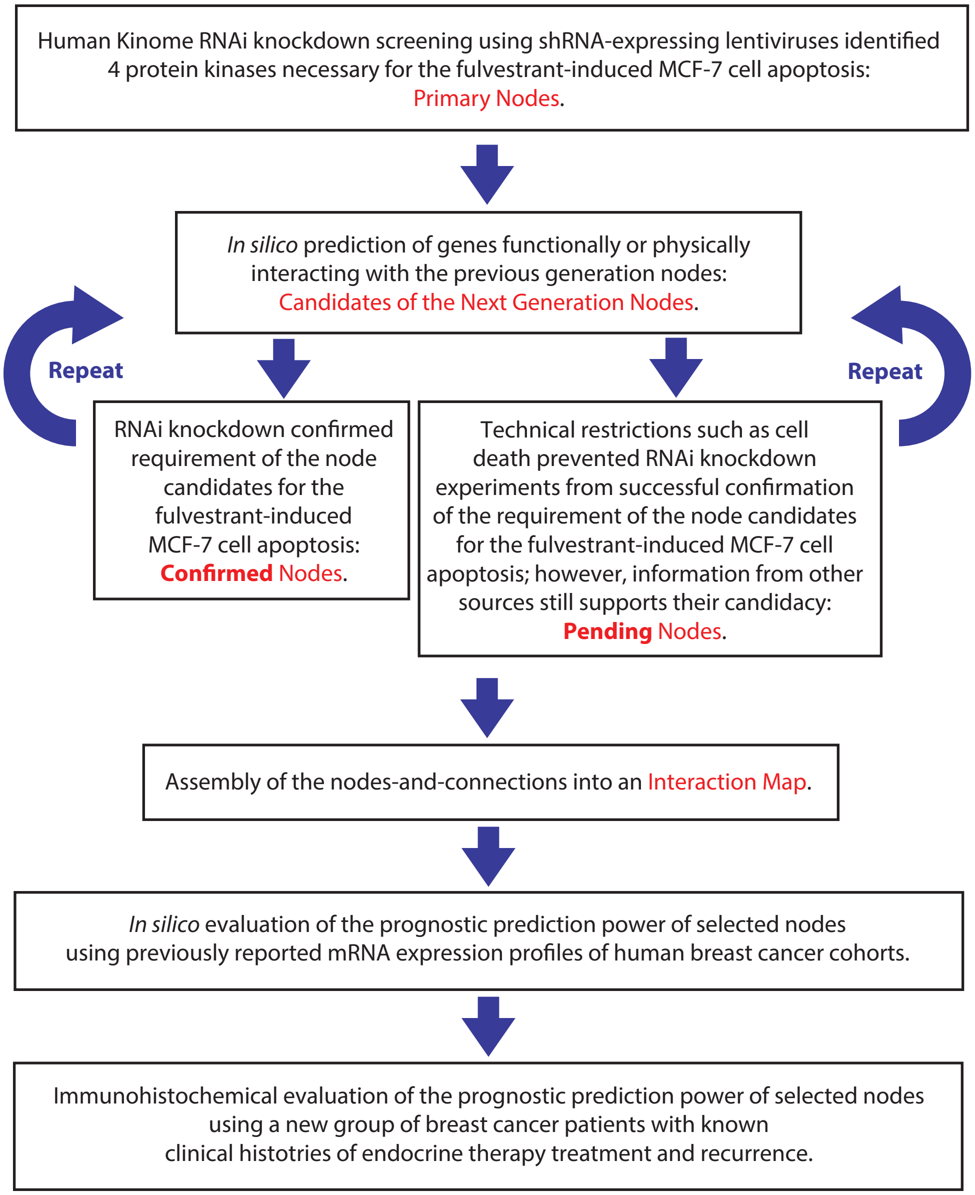


**Supplementary Figure 1.** Study overview. The initial human kinome RNAi knockdown screenings identified four protein kinases required for the fulvestrant-induced apoptosis of MCF-7 cells. Repeated *in silico* prediction of signaling proteins followed by confirmation with RNAi knockdown experiments generated an interaction map of molecules required for this fulvestrant action. *In silico* evaluations using mRNA expression profiles of previously reported breast cancer cohorts and immunohistochemical assessments supported the usefulness of the interaction map component nodes for prognostic prediction of breast cancer patients.
