# Supplementary material for: Nodes-and-connections RNAi knockdown screening: identification of a signaling molecule network involved in fulvestrant action and breast cancer prognosis
Source: Oncogenesis. 2015 Oct 19;4(10):e172–. doi: 10.1038/oncsis.2015.32 (PMC4632093; doi:10.1038/oncsis.2015.32)
Supplement: Supplementary Figure 2 [file oncsis201532x2.pdf]

A

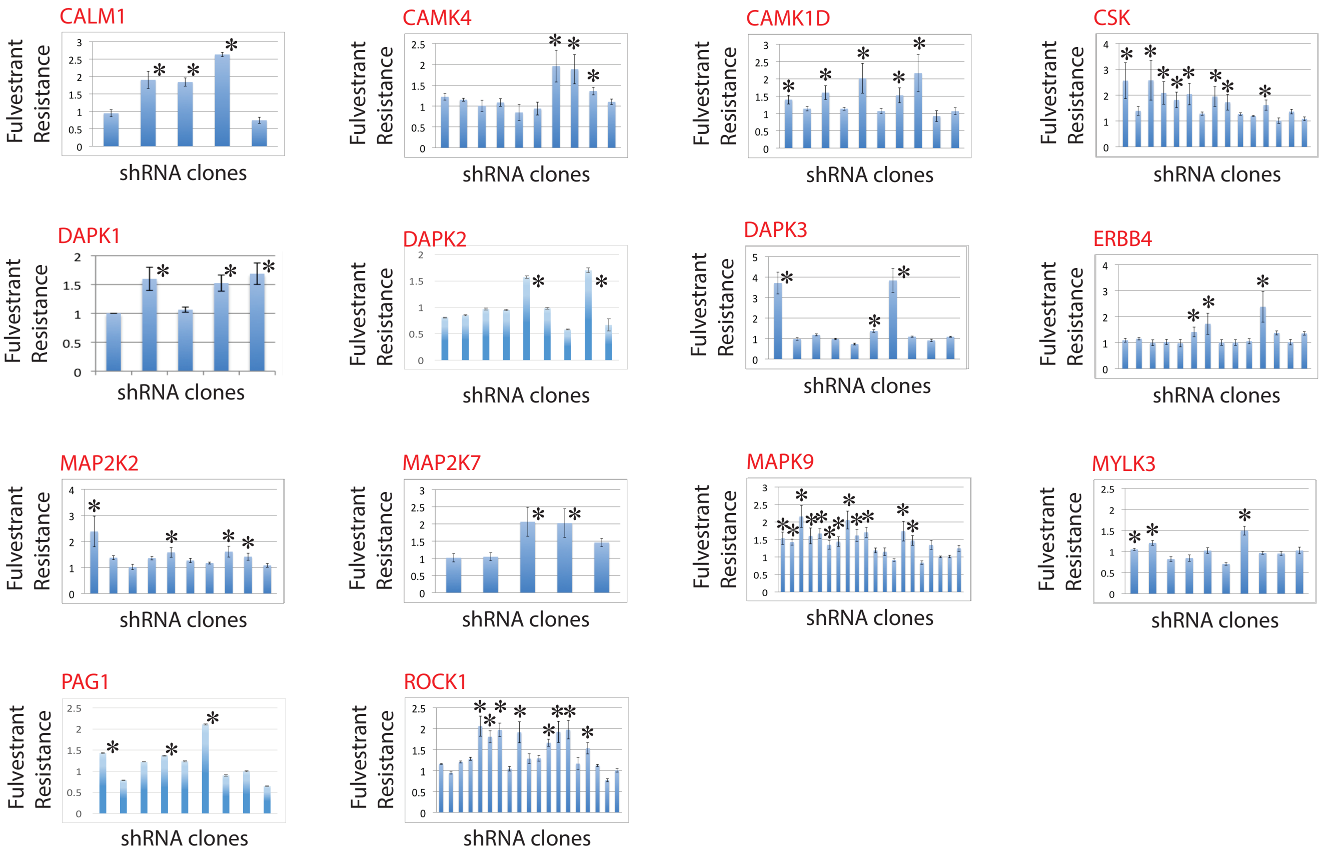

B

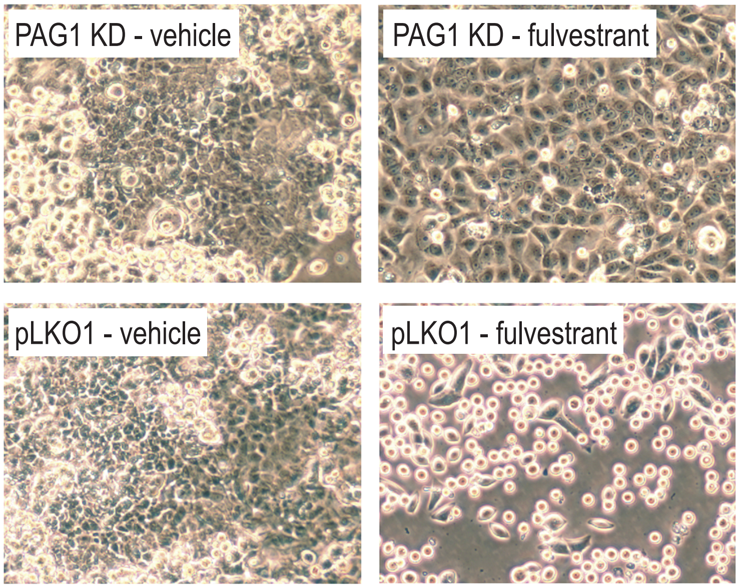

Supplementary Figure 2. (A) Validation of fulvestrant resistance of MCF-7 cells infected by lentiviruses expressing shRNA to nodal genes. Cells were infected with lentiviruses expressing shRNA species that target indicated genes and then exposed to 100 nM fulvestrant for 7 days followed by counting live cells. Relative fulvestrant resistance of cells infected with the pLKO.1 control lentivirus vector was defined to be 1.0. Each bar represents results of three independent experiments (mean ± SEM). Asterisk indicates statistically significant resistance ($p<0.05$, ANOVA). (B) Fulvestrant resistance of MCF-7 cells with PAG1 knockdown: Phase contrast microscopic images. Cells infected by lentiviruses expressing shRNA to PAG1 (PAG1 KD) or control lentivirus vector (pLKO1) were exposed to 100 nM fulvestrant or vehicle for 7 days. Vehicle-exposed cell cultures show dense and pileup appearance with or wihout PAG1 knockdown. Cells infected by control virus show massive fulvestrant-induced apoptosis. PAK1 knockdown cells show minimum sign of cell death although cell culture density is lower than vehicle-exposed control.
