# Supplementary material for: Nodes-and-connections RNAi knockdown screening: identification of a signaling molecule network involved in fulvestrant action and breast cancer prognosis
Source: Oncogenesis. 2015 Oct 19;4(10):e172–. doi: 10.1038/oncsis.2015.32 (PMC4632093; doi:10.1038/oncsis.2015.32)
Supplement: Supplementary Table 1 [file oncsis201532x3.pdf]

Supplementary Table 1
RNAi knockdown of genes required for the fulvestrant-induced MCF-7 cell death

Gene Symbol	Node Generation	TRC clone ID	shRNA sequence
DAPK3	1	TRCN0000055426	CGTTCACTACCTGCACTCTAA
		TRCN0000000519	CAACCCACGAATCAAGCTCAT
		TRCN0000009945	CGTTCACTACCTGCACTCTAA
ERBB4	1	TRCN0000001407	GCTAAGATAAAGACTGTGGAA
		TRCN0000039688	CAGTTCTCTGTGGTTCAGGAA
		TRCN0000039689	GCCCGTAATGTCTTAGTGAAA
MAP2K2	1	TRCN0000007005	CCAACATCCTCGTGAACTCTA
		TRCN0000007007	TGGACTATATTGTGAACGAGC
		TRCN0000007008	CTGGACTATATTGTGAACGAG
		TRCN0000199043	CGACAGCGCATGCAGGAACGG
BIK	1	TRCN0000033524	CCACACTTAAGGAGAACATAA
		TRCN0000033525	ACTGAGGACATCAGGGATGTT
		TRCN0000033526	CTTGGCATGACTGACTCTGAA
		TRCN0000033527	CTTCGATTCTTTGGAATGCAT
		TRCN0000033528	TGGACGGTTTCACCACACTTA
		TRCN0000432225	TCTTGATGGAGACCCTCCTGT
		TRCN0000436254	GCGGCCTGCTGCTGTTATCTT
DAPK1	2	TRCN0000000983	CCACGTCGATACCTTGAAATT
		TRCN0000000984	CGGCACCTCTTACAATTCCAT
		TRCN0000000985	CGACATCCAGAACGCTTATTT
DAPK2	2	TRCN0000001722	TGCTCCAGAAATTGTGAACTA
		TRCN0000196486	GAAGATGGAGTTGAATTTAAG
ROCK1	2	TRCN0000002163	GCCAGCAAAGAGAGTGATATT
		TRCN0000121092	CGGGTTGTTCAGATTGAGAAA
		TRCN0000121093	GCACCAGTTGTACCCGATTTA
		TRCN0000121095	GCATTCCAAGATGATCGTTAT
		TRCN0000121314	CGATTCTATACTGCAGAAGTA
		TRCN0000121315	CGGATTCTACAAGTGTTGCTA
		TRCN0000121316	GAGGAGGAGATCAGTAATCTT
		TRCN0000195202	CGATCGTCTCTAGGATGATAT
TP53	2	TRCN0000003753	CGGCGCACAGAGGAAGAGAAT
		TRCN0000003754	TCAGACCTATGGAAACTACTT
		TRCN0000003755	GTCCAGATGAAGCTCCCAGAA

		TRCN0000003756	CACCATCCACTACAACTACAT
		TRCN0000342261	GAGGGATGTTTGGGAGATGTA
		TRCN0000342259	GTCCAGATGAAGCTCCCAGAA
		TRCN0000342334	CACCATCCACTACAACTACAT
		TRCN0000342335	CGGCGCACAGAGGAAGAGAAT
		TRCN0000010814	GAGGGATGTTTGGGAGATGTA
MYLK3	3	TRCN0000220105	TGCAGAAATACATAGCTCAAA
		TRCN0000199722	GCAGAGCATGTGCCGAGACAT
		TRCN0000199527	GACCTCCATCTCTGCGGGTTA
CAMK1D	3	TRCN0000001752	TGCTGTGAAGTGTATCCCTAA
		TRCN0000001753	AGAATGAGATAGCCGTCCTGA
		TRCN0000001754	AGGCGGAATATGAGTTTGACT
		TRCN0000001756	TGATGGAGAAGGACCCGAATA
		TRCN0000195531	CGGAGTGATTGCCTACATCTT
CAMK4	3	TRCN0000009961	TGGTCCTAGAACTCGTCACAG
		TRCN0000009962	GATATTACAGTGAGCGAGATG
		TRCN0000009963	GAGGCGATCAGTTCATGTTCA
MAPK9	3	TRCN0000001012	GAGCAGTTAGAGTAGGTGAAT
		TRCN0000001013	GATGTGTATTTGGTTATGGAA
		TRCN0000001014	CTGTGAGGAATTATGTCGAAA
		TRCN0000001015	AGGGATTGTTTGTGCTGCATT
		TRCN0000001016	GTTATTCACATGGAGCTGGAT
		TRCN0000000944	GATGTGTATTTGGTTATGGAA
		TRCN0000000945	CTGTGAGGAATTATGTCGAAA
		TRCN0000000946	AGGGATTGTTTGTGCTGCATT
		TRCN0000000947	GTTATTCACATGGAGCTGGAT
		TRCN0000010279	ACTGTGAGGAATTATGTCGAA
		TRCN0000010280	GCGTCACCCATACATCACTGT
		TRCN0000196315	GCTTCTGAAGTTATCTCTTAA
PAG1	4	TRCN0000123271	CCTGTAATGATCTCTATGCTA
		TRCN0000123273	TGATCTCTATGCTACTGTTAA
		TRCN0000434656	TTAAGCTTCTGGACGAGAATG
MAP2K7	4	TRCN0000001079	CACAGGAAGAGACCAAAGTAT
		TRCN0000001080	CTACAAGAACTGCAAGACGGA
CALM1	4	TRCN0000037570	AGAAGCTGAATTGCAGGATAT
		TRCN0000037571	GACGGACAAGTCAACTATGAA
		TRCN0000037572	CGTGAGGCATTCCGAGTCTTT
CSK	5	TRCN0000199018	CCACTAAGTCTGACGTGTGGA

TRCN0000199031	CCGTCTCTCTTGGACCCACCT
TRCN0000199500	GCCCGCAGTCTATGAAGTCAT
TRCN0000000804	CGAGGAGGTGTACTTTGAGAA
TRCN0000000806	GAGAAGGGCTACAAGATGGAT
TRCN0000010007	GTACCAAACTCAGCCTCATGC
TRCN0000010008	ACTACACCTCAGACGCAGATG
TRCN0000010009	GTACGCGCCTCATTAAACCAA
